# Intermittency in Transitional Shear Flows

**DOI:** 10.3390/e23030280

**Published:** 2021-02-26

**Authors:** Yohann Duguet

**Affiliations:** LIMSI-CNRS, University Paris-Saclay, F-91400 Orsay, France; duguet@limsi.fr

The study of the transition from a laminar to a turbulent flow is as old as the study of turbulence itself. Since the seminal pipe flow experiments of O. Reynolds at the end of the XIXth century, it is understood that turbulent velocity fluctuations do not emerge in a regular way. Instead they appear via intermittent bursts of activity in an otherwise laminar environment [1]. A series of experiments carried out in the last century have demonstrated, in most incompressible fluid flows occurring near solid walls, the existence of a transitional range at the onset of the turbulent regime. This specific parameter range corresponding to low velocities has been labelled by hydraulic engineers the “uncertainty zone” because of the difficulty to perform either deterministic nor statistical prediction of the flow. Only around the end of the XXth century did researchers begin to understand that laminar-turbulent intermittency features a higher degree of organization, in the statistical sense, than previously thought. Yet, technical limitations as well as finite-size effects have made rigorous investigation notoriously difficult because of the different length scales and timescales involved.

The last decade has witnessed a quickly growing number of decisive contributions, made possible by the huge progress in computational power, in experimental measurements and in visualization techniques. Theoretical progress, notably due to an exciting analogy with the thermodynamical formalism of phase transitions, has motivated most of these recent advancements [2]. It is now well established that the transitional range, parameterized by the so-called Reynolds number proportional to the fluid velocity, features a regime of laminar-turbulent patterning. It has been advanced for several decades that the lower transitional range features a continuous transition belonging to the universality class of directed percolation. The hydrodynamical and statistical organization of these coherent structures considered individually remain however not well understood. Finally, there are open issues about to how universal these results are, given the variety of different fluid flow cases.

The goal of the present special issue is to give an up-to-date overview of this cross-disciplinary topic. It contains nine original research articles written by specialists from the most active research teams in the field.

No less than three detailed experimental investigations of the transitional regime of Taylor-Couette and plane channel flow are part of this special issue. The study by K. Avila and B. Hof [3] establishes with minimal finite-size effects that the turbulent fraction evolves continuously with the Reynolds number, rather than discontinuously as often believed. The two experimental investigations of channel flow by J. Liu et al. [4] and by M. Agrawal et al. [5] contain a rich and complementary database on friction fluctuations in channel flow.

A series of careful direct numerical studies explore the dynamics of individual coherent structures at the onset of turbulence, in possible connection with the directed percolation regime expected theoretically. Morimatsu and Tsukahara [6] focus on the mechanisms leading localized turbulent structures in annular Couette-Poiseuille flow to split into two. Takeda et al. [7] verify the existence of a critical range of annular Couette flow using artificial extensions of numerical domains. X. Xiao and B. Song [8] focus on the dynamics of localized turbulent bands at the onset of turbulence in plane channel flow. 

The upper transitional range of channel flow features clear oblique patterns, as investigated numerically by P. Kashyap et al. [9]. They demonstrate there an unusual link across the transitional and full-fledged turbulent regime via high-order statistics of the wall shear stress. Low-order modelling covering all these intermittent sub-regimes of plane channel flows is also considered in the contribution by P. Manneville and M. Shimizu, based on the simple concept of cellular automata [10].

Eventually, the special issue includes an original extension of the intermittency concepts to pulsatile flows by D. Feldmann et al. [11]. Possible applications to cardiovascular diseases up a new line of research in connection to biological applications. 

This special issue is meant to represent a snapshot of the field at the beginning of this new decade. It aims at fostering interaction and debates, and not at all to close possible debates. Overall, the associated articles represent a timely perspective of the current research in hydrodynamics as well as, more generically, in complexity science. They suggest that the field of intermittent hydrodynamics has now reached the age of maturity.
